# Gestational Dynamics of Hofbauer Cells in the Human Placenta: Distribution, Morphology, and Immunophenotype

**DOI:** 10.3390/jdb14020026

**Published:** 2026-06-02

**Authors:** Sanja Jovičić, Ivan R Nikolić, Ljiljana Božić, Marko Jović, Dina Kapić, Ranko Škrbić

**Affiliations:** 1 Center for Biomedical Research, Faculty of Medicine, University of Banja Luka, 78000 Banja Luka, Bosnia and Herzegovina; ljiljana.bozic@med.unibl.org; 2 Department of Histology and Embryology, Faculty of Medicine, University of Banja Luka, 78000 Banja Luka, Bosnia and Herzegovina; 3 Department of Histology and Embryology, Faculty of Medicine, University of Niš, 18000 Niš, Serbia; inikolic@junis.ni.ac.rs (I.R.N.); marko.jovic@medfak.ni.ac.rs (M.J.); 4 Department of Microbiology, Faculty of Medicine, University of Banja Luka, 78000 Banja Luka, Bosnia and Herzegovina; 5 Department of Histology and Embryology, Faculty of Medicine, University of Sarajevo, 71000 Sarajevo, Bosnia and Herzegovina; dina.kapic@mf.unsa.ba; 6 Department of Pharmacology and Toxicology, Faculty of Medicine, University of Banja Luka, 78000 Banja Luka, Bosnia and Herzegovina; ranko.skrbic@med.unibl.org; 7 Department of Pathologic Physiology, First Moscow State Medical University I.M. Sechenov, 119435 Moscow, Russia; 8 Department of Medical Sciences, Academy of Sciences and Arts of the Republic of Srpska, 78000 Banja Luka, Bosnia and Herzegovina

**Keywords:** Hofbauer cells, human placenta, non-M1 macrophage phenotype, proliferative index

## Abstract

Background: Hofbauer cells (HBCs) are the only immunocompetent cells within the stroma of chorionic villi and play a key role in immune regulation and placental development throughout gestation. Their phenotype, abundance, and proliferative activity change in accordance with the needs of the fetoplacental unit. Methods: Thirty healthy human placentas across all three trimesters were analyzed. Samples were processed using standard histological protocols and immunohistochemically stained with CD45, CD68, CD86, and Ki-67 markers. Morphometric analysis was performed to determine the following parameters: percentage of HBCs, numerical areal density, and proliferative index. Results: HBCs were immunoreactive for CD45 and CD68, while CD86 immunoreactivity was not observed in any trimester. The proportion of HBCs was highest in the second trimester and lowest in the third. Numerical areal density was highest in the second trimester (22.21 ± 3.86) and lowest in the first (8.27 ± 4.18). The proliferative index was highest in the first trimester (82.45 ± 10.19%), decreased significantly in the second, and was completely absent in the third trimester. Conclusions: During physiological placental development, Hofbauer cells maintain a predominantly non-M1 macrophage phenotype, accompanied by a gradual reduction in proliferative activity.

## 1. Introduction

Hofbauer cells (HBCs) are placental macrophages of fetal origin that are present throughout gestation [1,2]. Macrophages are mononuclear phagocytic cells that play essential roles in innate and adaptive immunity. In addition to their defensive functions, macrophages contribute to tissue homeostasis, development, and repair. They represent a heterogeneous immune cell population capable of dynamically adapting their phenotypic and functional properties in response to changes in the local microenvironment [3].

Under physiological conditions, HBCs are the only immunocompetent cells within the stroma of placental chorionic villi. As cells of fetal origin, they contribute to fetal protection through phagocytosis, antigen presentation, and cytokine secretion [4,5]. Their activity supports immune tolerance at the maternal–fetal interface and helps maintain a controlled balance between pro-inflammatory and anti-inflammatory processes within the placenta. Phenotypically, HBCs are commonly classified into two major macrophage subtypes: pro-inflammatory M1 and anti-inflammatory M2 macrophages. The M1 subtype is characterized by the production of cytokines such as interleukin-6 (IL-6), interleukin-12 (IL-12), and tumor necrosis factor (TNF), whereas M2 macrophages are associated with suppression of inflammatory responses, promotion of angiogenesis, and facilitation of tissue repair [6,7,8]. Based on surface marker expression and functional characteristics, most studies indicate that this cell population predominantly exhibits an M2-like phenotype, consistent with their anti-inflammatory and reparative roles in the placenta [7,8,9].

The localization of HBCs in close proximity to fetal blood vessels and trophoblast cells further supports their involvement in angiogenesis, vasculogenesis, and placental homeostasis [2,10]. Within the stroma of chorionic villi, these cells show characteristic morphology and distribution, which in some cases allows their recognition on H&E-stained sections. However, particularly in early-gestation placentas, their dense arrangement among other stromal cells makes reliable identification by routine staining difficult. Therefore, specific immunohistochemical markers are required for accurate detection and quantification. Morphologically, they display considerable variability, appearing round, oval, spindle-shaped, or triangular, with an eccentrically positioned nucleus and cytoplasm rich in vacuoles and lysosomes. Their cell surface frequently exhibits lamellipodia, microvilli, and membrane ruffles, reflecting high phagocytic activity. The size and number of cytoplasmic vacuoles vary during gestation, being relatively small in early and late pregnancy and more prominent during mid-gestation [11].

Despite extensive investigation, the origin and differentiation pathways remain incompletely understood. Early HBCs, detected up to the fourth week of pregnancy, are thought to originate from mesenchymal cells of the parietal layer of the extraembryonic mesoderm. In contrast, later HBC populations appear during the second wave of hematopoiesis, independent of hematopoietic stem cells, and arise from the visceral layer of the extraembryonic mesoderm of the secondary yolk sac. These cells exhibit features of tissue-resident macrophages, including long-term persistence and self-renewal capacity [9,12].

Although the immunophenotypic properties of HBCs have been described in previous studies, important gaps remain regarding their quantitative dynamics during normal human pregnancy. In particular, systematic trimester-resolved analyses of HBC density, proliferation, morphology, and spatial distribution in physiological placentas are limited. Most available studies focus on first-trimester or term placentas, whereas mid-gestational placental development—a critical phase of placental maturation—remains insufficiently characterized. To address these gaps, the present study applies a standardized morphometric and immunohistochemical approach across all three trimesters of pregnancy, enabling a comparable assessment of this placental macrophage population dynamics throughout gestation. By integrating quantitative, spatial, and phenotypic analyses, this study provides a comprehensive characterization of the morphological and functional adaptations of this cell population across gestation in morphologically preserved placentas.

The aim of this study was to define the phenotypic profile and examine the gestational dynamics of HBCs in morphologically preserved human placentas across gestation, with particular emphasis on their morphological, topographical, immunohistochemical, and quantitative characteristics across different stages of pregnancy.

## 2. Materials and Methods

### 2.1. Tissue Sampling

The study was approved by the Ethics Committee of the Faculty of Medicine, University of Banja Luka, Bosnia and Herzegovina (approval no. 18/4.167/21), and conducted in accordance with the principles of the Declaration of Helsinki (latest revision). A total of 30 healthy human placentas were included: 10 from the first trimester (gestational age 7–12 weeks), 10 from the second trimester (14–25 weeks), and 10 from the third trimester (26–37 weeks), (Table 1).

First-trimester placentas were collected at the Institute of Pathology, University Clinical Center of the Republic of Srpska (UCC RS), from tubal pregnancies submitted for histopathological examination due to suspected fallopian tube rupture. Although intratubal pregnancies occur at an ectopic site, embryonic and placental development remains morphologically preserved until approximately the 11th–12th week of gestation [13], while placental tissue obtained from elective abortions was excluded due to potential mechanical disruption. Among the second-trimester samples obtained following pregnancy termination, Down syndrome was the most frequent indication, whereas Edwards syndrome and Patau syndrome were each represented by a single case. Samples from pregnancies terminated because of isolated fetal organ anomalies, including cardiovascular malformations, were excluded, since such conditions may be associated with alterations in placental morphology, particularly in vascular structures. These samples were also processed at the Institute of Pathology after routine histopathological examination. Third-trimester placentas were collected at the Clinic of Gynecology and Obstetrics after vaginal delivery.

All analyzed samples were obtained from placentas of healthy, non-smoking pregnant women younger than 35 years, with no history of hypertension, diabetes mellitus, or infectious diseases during pregnancy, and with no documented medication use, except for micronized progesterone administered in the third trimester in cases complicated by cervical insufficiency [14]. Only samples with well-preserved placental morphology and no signs of inflammation, hemorrhage, or structural abnormalities were included.

### 2.2. Tissue Processing

All samples contained the full thickness of the placenta, from the chorionic to the basal plate. After 24 h of fixation in 4% formaldehyde, tissue samples underwent automated processing in a tissue processor (Leica TP1020, Wetzlar, Germany) and were subsequently embedded in paraffin blocks. Sections were cut at a thickness of 4 µm and stained using routine hematoxylin and eosin staining, as well as immunohistochemical staining with the following antibodies: anti-CD45 (Clone SPM946, dilution 1:100, Santa Cruz Biotechnology, Dallas, TX, USA), anti-CD68 (ab192847, dilution 1:100, Abcam, Cambridge, CB2 0AX, UK), anti-CD86 (ab269587, dilution 1:200, Abcam, Cambridge, CB2 0AX, UK), and anti-human Ki-67 (clone MIB-1, Dako, Ready to Use, 5301 Stevens Creek Blvd, Santa Clara, CA, USA). Antigen retrieval was performed by heating the tissue sections in citrate buffer (pH 6) for 20 min. Endogenous peroxidase activity was blocked with 3% hydrogen peroxide, while nonspecific background staining was blocked using UltraVision Block. Primary antibodies were incubated at room temperature for 30 min. The detection was performed using a secondary antibody conjugated with horseradish peroxidase-labeled polymer. Diaminobenzidine was then applied to visualize peroxidase activity following the manufacturers guidelines (HRP/DAB IHC detection system, ab236466, Abcam, Cambridge, CB2 0AX, UK). CD45 was used as a marker of hematopoietic cells and HBCs. CD68 served as a well-established pan-macrophage marker expressed in both M1 and M2 macrophages. CD86 staining was used to assess (and exclude) an M1-like phenotype in HBCs. Finally, Ki-76 immunostaining was performed to evaluate the proliferative activity of HBCs, as Ki-76 is expressed during the active phases of the cell cycle. Analysis of the obtained samples was performed using a binocular microscope (Leica DM 6000, Wetzlar, Germany) equipped with a Leica DFC310FX camera. The intensity of immunoreactivity for CD45, CD68, CD86, and Ki-67 was assessed semi-quantitatively as absent (−), low (+), moderate (++), or high (+++).

### 2.3. Morphometric Analysis

Morphometric analysis was performed using QuPath software (version 0.5.6). Microphotographs captured at ×400 magnification were analyzed. The HBCs were identified according to their characteristic localization within the villous stroma, morphological features, and the expression of specific surface markers confirmed by immunohistochemistry. The following parameters were evaluated: the percentage of HBCs relative to the total number of stromal cells within each chorionic villus, numerical areal density (N_A_), and the proliferative index.

The percentage of immunoreactive HBCs was calculated using the formula:

N (%) = (N × 100)/Σ, where N represents the number of immunoreactive cells and *Σ* the total number of stromal cells.

Numerical areal density (N_A_) was defined as the number of examined cells per unit area of the chorionic villus. The villous area (µm^2^) was first measured, and N_A_ was calculated using the formula: N_A_ = N/A, where N is the number of examined cells and *A* is the villous area [15].

The proliferative index was calculated as the percentage of Ki-67-positive nuclei relative to the total number of cells in each microscopic field. Final values were expressed as the mean across all analyzed fields [16].

The number of analyzed microscopic fields (N) was determined using the formula:

N = (20 × SD/X)^2^, where SD represents the standard deviation and X the mean value obtained from a pilot study conducted on 20 microscopic fields [17]. Accordingly, 25 microscopic fields were analyzed for each tissue section at ×400 magnification.

### 2.4. Statistical Analysis

R software (version 4.2.3) was used for statistical analysis. Descriptive statistics were used to organize and summarize the data. Numerical areal density (N_A_), Hofbauer cell counts, and the distributions of Ki-67 immunoreactivity (proliferative index), were assessed for normality using skewness, kurtosis, and the Shapiro–Wilk test. Student’s *t*-test was applied to compare means between two groups, while one-way analysis of variance (ANOVA) was used to evaluate differences among more than two groups. A *p*-value ≤ 0.05 was considered statistically significant.

## 3. Results

### 3.1. Morphological Findings

**Figure 1:**
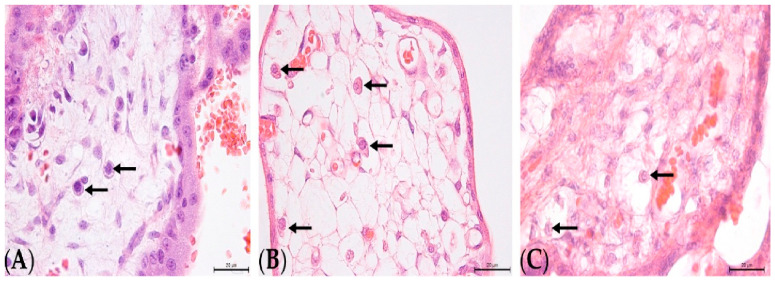
Morphological characteristics of Hofbauer cells in human placentas during the first, second, and third trimesters (H&E staining; magnification x630; scale bar = 20 µm). (**A**) Hofbauer cells (black arrows) within the stroma of immature intermediate chorionic villi in a first-trimester placenta (10 weeks of gestation). The cells are predominantly round, with an eccentrically positioned round nucleus and abundant cytoplasm. (**B**) Hofbauer cells (black arrows) within the stromal channels of mature intermediate chorionic villi in a second-trimester placenta (20 weeks of gestation). The cells show marked polymorphism, appearing round, oval, or spindle-shaped, with centrally positioned nuclei, abundant cytoplasm, and an average size of up to 15 µm. (**C**) Hofbauer cells (black arrows) within the stromal channels of mature intermediate chorionic villi in a third-trimester placenta (30 weeks of gestation). The cells are predominantly round, approximately 10 µm in diameter, and contain slightly less cytoplasm than those observed in the second trimester.

Hofbauer cells were present in the stroma of various types of chorionic villi, starting from the ninth week of gestation up to the thirty-seventh week of gestation. They were located in the stroma of immature and mature intermediate villi, as well as terminal chorionic villi, where they predominantly resided within stromal channels. Morphologically, these cells displayed polymorphism in terms of size, shape, and degree of immunoreactivity. Hofbauer cells were most commonly oval in shape, although they could also appear triangular, polygonal, or elongated (fibroblast-like). Their diameter ranged from 10 to 15 μm (Figure 1). It was observed that Hofbauer cells in third-trimester placentas had a smaller diameter compared to those in the first and second trimester, which may have indicated morphological maturation and functional adaptation during gestation.

### 3.2. Immunohistochemical Findings

**Figure 2:**
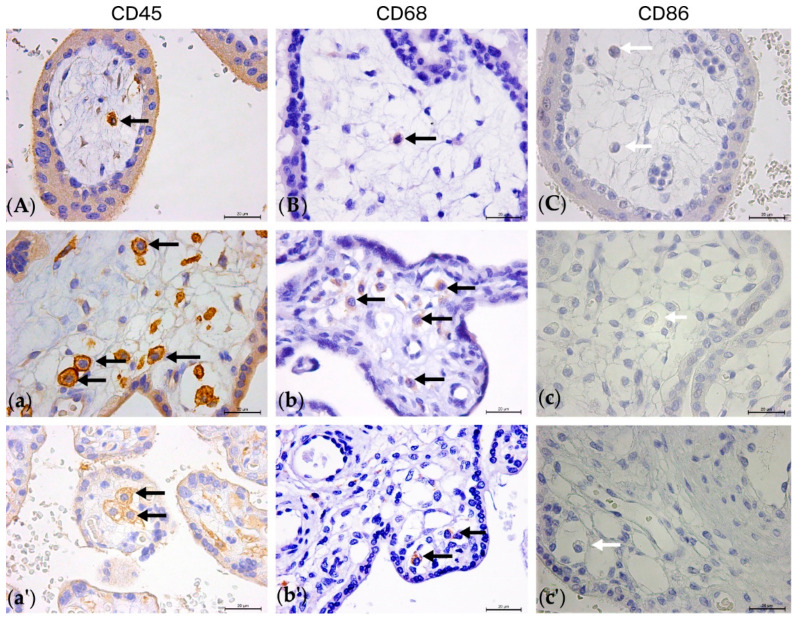
Immunohistochemical features of Hofbauer cells (HBCs) within the stroma of chorionic villi during the first (**A**–**C**), second (**a**–**c**), and third (**a′**–**c′**) trimesters of pregnancy (IHC staining: anti-CD45, anti-CD68, and anti-CD86; magnification x630; scale bar = 20 µm)**.** CD45 (**A**,**a**,**a′**): Hofbauer cells (black arrows) show strong CD45 positivity (+++) in the villous stroma throughout all gestational periods, with intense membranous and cytoplasmic staining. CD68 (**B**,**b**,**b′**): Strong CD68 positivity (+++) is present in Hofbauer cells (black arrows) in all three trimesters, consistent with their macrophage phenotype. CD86 (**C**,**c**,**c′**): Hofbauer cells (white arrows) show no CD86 expression in any trimester, supporting the absence of M1-polarized macrophages under morphologically preserved placentas.

Hofbauer cells demonstrated strong immunoreactivity for CD45 and CD68 (+++) in all examined gestational periods, confirming their macrophage phenotype (Figure 2A,a,a′,B,b,b′). In contrast, no CD86-immunoreactive Hofbauer cells were detected in any trimester (−), indicating the absence of an M1-like macrophage population in morphologically preserved placentas (Figure 2C,c,c′).

**Figure 3:**
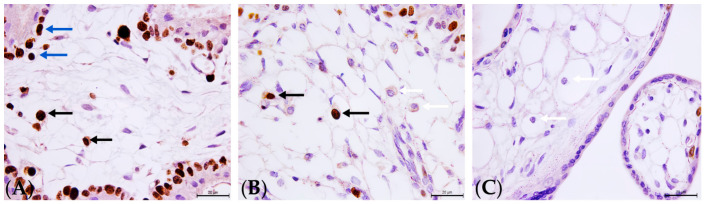
Immunohistochemical expression of Ki-67 in human placental villi during the first (**A**), second (**B**), and third (**C**) trimesters of pregnancy (IHC staining: anti-Ki-67; magnification x630; scale bar = 20 µm). (**A**) In the first trimester (10 weeks of gestation), HBCs show strong Ki-67 positivity (+++) (black arrows), indicating high proliferative activity. Hematopoietic stem cells and cytotrophoblast cells also exhibit strong Ki-67 expression (blue arrows), reflecting their high proliferative potential. (**B**) In the second trimester (20 weeks of gestation), a mixed population of HBCs is present, with both Ki-67-positive (+++) cells (black arrows) and Ki-67-negative cells (white arrows). (**C**) In the third trimester (30 weeks of gestation), HBCs are uniformly Ki-67-negative (white arrows), reflecting the loss of proliferative potential in late gestation.

In the first trimester, Hofbauer cells exhibited strong Ki-67 immunoreactivity (+++), reflecting high proliferative activity. In the second trimester, Ki-67 immunoreactivity was reduced, with both positive and negative cells observed. In the third trimester, Hofbauer cells showed no Ki-67 immunoreactivity, indicating the absence of proliferative activity at this stage (Figure 3).

### 3.3. Morphometric Analysis

Quantitative analysis showed that HBCs were relatively sparse compared to the total cellular population within the stroma of chorionic villi. In first-trimester placentas, they accounted for 8.16 ± 4.57% of all stromal cells; in the second trimester 8.67 ± 1.62%; while in the third trimester their proportion was the lowest, at 5.65 ± 1.13%. Statistical analysis revealed a significant difference in the number of HBCs among trimester groups (*p* ≤ 0.05) (Table 2). Post hoc analysis revealed that the mean value of HBCs numbers differed significantly between the second and third trimesters (*p* = 0.0003), whereas no significant differences were observed between the first and second trimesters (*p* = 0.71) or between the first and third trimesters (*p* = 0.16).

The highest values of numerical areal density were recorded in the second trimester, reaching 22.21 ± 3.86. Slightly lower values were observed in the third trimester (10.72 ± 4.54), whereas the lowest values were found in the first trimester (8.27 ± 4.18). Statistical analysis demonstrated a highly significant difference in the numerical areal density of HBCs between trimesters (*p* < 0.001) (Table 2). Post hoc analysis revealed highly significant differences in mean values of numerical areal density between the first and second trimesters (*p* < 0.001), and between the second and third trimesters (*p* < 0.001), whereas no statistically significant difference was observed between the first and third trimesters (*p* = 0.32).

The highest proliferative potential of HBCs was observed in the first trimester, with the greatest proportion of Ki-67-positive nuclei (82.45 ± 10.19%). A marked decrease in the proliferative index was noted in the second trimester (56.7 ± 11.89%), indicating a progressive reduction in proliferative activity as gestation advances. In the third trimester, no Ki-67 immunoreactivity was detected, suggesting that Hofbauer cells no longer exhibit proliferative activity in late pregnancy (Figure 3). Statistical analysis confirmed a significant difference across trimesters (*p* ≤ 0.05) (Table 2).

## 4. Discussion

The placenta is a dynamic organ that undergoes continuous morphological and functional changes throughout gestation in order to meet the increasing demands of the developing fetus [18]. These processes include tissue remodeling, angiogenesis, and tightly regulated immunological interactions that together maintain immune balance at the maternal–fetal interface. Although trophoblast cells have traditionally been regarded as the primary regulators of these adaptations, accumulating evidence indicates that HBCs also play an important and multifaceted role in placental physiology and immunology [19,20]. Previous studies have demonstrated that these cells contribute to immune regulation within the placenta and are involved in physiological fetal development. In the present study, we focused on the phenotypic characteristics, distribution, and proliferative activity of HBCs from morphologically preserved placentas across gestation.

Their morphology may vary with gestational age and may also be influenced by maternal conditions, including excessive body weight and diabetes. Shook et al. reported that increased maternal body weight was associated with larger, more rounded, and more numerous HBCs [21]. In diabetic pregnancies, they have been described as more spindle-shaped and irregular, with increased accumulation of apoptotic bodies in the villous stroma. These findings suggest functional plasticity and may reflect a shift toward a more pro-inflammatory, M1-like phenotype. Fetal sex may further modulate this response, with female fetuses showing a greater ability to preserve a stable and tolerogenic phenotype [22]. In our study, the smaller diameter observed in early gestation may be related to their compact arrangement within the villous stroma, whereas the larger diameter in the second trimester may reflect freer distribution within stromal spaces and greater morphological expansion. The subsequent reduction in late gestation may be associated with maturational changes and cellular aging.

Our findings demonstrate that Hofbauer cells in morphologically preserved placentas express CD45 and CD68, whereas CD86 expression was not detected. This immunophenotypic profile is consistent with predominantly non-M1 macrophage phenotype, which is generally associated with anti-inflammatory and reparative functions; however, a more robust confirmation of this functional polarization would require the evaluation of additional markers. These observations are in agreement with previous reports describing a predominance of M2-polarized HBCs in morphologically preserved placentas, in contrast to studies showing increased M1 polarization in pathological conditions such as preeclampsia [7,23]. This phenotype is compatible with the proposed role of Hofbauer cells in maintaining immune tolerance at the maternal–fetal interface, as well as in extracellular matrix remodeling and the regulation of angiogenesis [24,25]. The persistence of predominantly non-M1 macrophage phenotype across all trimesters suggests their stable functional specialization during normal pregnancy [26]. This phenotype likely contributes to the establishment of a placental microenvironment that limits excessive inflammatory responses and supports physiological gestation. The absence of CD86 expression further underscores the anti-inflammatory character of these cells and is consistent with their proposed role in maintaining immune balance at the maternal–fetal interface [27].

Most authors report that HBCs can be identified in placental tissue obtained after elective abortions as early as 18 days after conception [7,28]. In the present study, however, this cell population was observed only in samples from the ninth week of gestation. The detected CD45 expression supports the hypothesis that early HBCs originate from embryonic hematopoietic progenitors, likely arising during the second wave of hematopoiesis from yolk sac or fetal liver sources [29,30,31,32]. As CD45 is also expressed by hematopoietic stem cells, it is possible that some of the detected cells may also originate from this population, although this would need to be confirmed in further studies using additional markers.

Morphometric analysis revealed that HBCs were most abundant during the second trimester. Their numbers increased from the ninth week of gestation, reached a maximum in mid-gestation, and declined toward term. Data on trimester-specific distribution, particularly during the second trimester, remain limited; however, available studies are generally consistent with the observed pattern [11,28,30,33]. The increased abundance during mid-gestation likely reflects intense placental growth, differentiation, villous remodeling, angiogenesis, and the need for enhanced immune regulation. The subsequent decrease in the third trimester may be related to progressive maturation of the fetal immune system, including the establishment of bone marrow hematopoietic activity [34,35]. Given their predominantly non-M1 phenotype and proposed roles in angiogenesis, stromal remodeling, and villous development, the reduced number in late gestation may also indicate that the placenta has largely completed its structural and functional maturation by this stage.

Analysis of proliferative activity demonstrated that HBCs exhibit the highest proliferative potential during the first trimester, with a progressive decline in the second trimester and complete absence of proliferative activity in the third trimester. This pattern is consistent with the rapid growth and vascular remodeling characteristic of early pregnancy, followed by a transition toward a more stable and functionally mature placental state in later gestation [2,30]. The high proliferative index observed in the first trimester may therefore reflect the active expansion during early placental development, contributing to the establishment of an adequate number of these cells in subsequent stages of pregnancy, particularly during the second trimester. As gestation progresses, the increase in placental tissue volume and ongoing remodeling of chorionic villi may require expansion of the resident fetal macrophage population. This interpretation is further supported by the strong Ki-67 expression observed in cytotrophoblastic cells during early gestation. Increased proliferative activity may also occur under pathological conditions, including viral infections, as suggested by Schwartz et al. However, the proliferative index values reported in that study were considerably higher in the second and third trimesters than those observed in our study [36]. It should also be noted that first-trimester data were not included, which limits direct comparison with our findings.

A key limitation of the present study is the restricted availability of placental tissue in early pregnancy, since first- and second-trimester samples are obtainable only through clinically indicated procedures or elective terminations. This should be considered when interpreting the developmental changes observed across gestation.

## 5. Conclusions

The present study demonstrates that Hofbauer cells are consistently present in the placental villous stroma from the ninth week of gestation and maintain a predominantly non-M1 macrophage phenotype, characterized by CD45 and CD68 expression in the absence of CD86. Their abundance varies across gestation, peaking in the second trimester, while their proliferative activity appears to be restricted to early pregnancy and declines with advancing gestation. These findings support the view that HBCs play an important role in maintaining immune tolerance and placental homeostasis across gestation in morphologically preserved placentas, and they provide a foundation for future studies exploring their involvement in pathological conditions such as preeclampsia and intrauterine growth restriction.

## Figures and Tables

**Table 1:** Distribution of placental samples according to developmental period, weeks of gestational age, number of placentas, and maternal age.

Development Period	WGA	Placentas N	Maternal Age Mean ± SD
**First Trimester N = 10**	7	1	22.1 ± 2.55
	8	2	
	9	2	
	10	1	
	11	2	
	12	2	
**Second Trimester N = 10**	19	5	32.8 ± 2.74
	20	4	
	23 *	1	
**Third Trimester N = 10**	30 *	1	27.9 ± 3.17
	35 *	1	
	36	5	
	37	3	

WGA—weeks of gestational age; N—number of placentas at a given gestational age; SD—standard deviation; * preterm birth due to cervical insufficiency.

**Table 2:** Morphometric characteristics of Hofbauer cells in human placentas across the first, second, and third trimesters.

	Trimester	Mean	SD	*p*-Value
Proportion of HBCs (%)	First	8.16	4.57	
	Second	8.67	1.62	0.05 *
	Third	5.65	1.13	
Numeric areal density	First	8.27	4.18	<0.001 **
	Second	22.21	3.86	
	Third	10.72	4.54	
Proliferative index (%)	First	82.45	10.19	0.046 *
	Second	56.7	11.89	
	Third	0	0	

Data are expressed as frequency and mean ± SD; * statistically significant, *p* ≤ 0.05; ** highly statistical significance, *p* < 0.001.

## Data Availability

The original contributions presented in this study are included in the article. Further inquiries can be directed to the corresponding authors.
